# Experimental comparisons of optical coherence tomography-based versus angiography-based time-averaged wall shear stress estimations

**DOI:** 10.1007/s10554-026-03649-1

**Published:** 2026-02-20

**Authors:** Jarka Naser, Nicholas A. Fogell, Miten Patel, Pan Yang, Mayurey Kalaravy, Fotios Savvopoulos, Rob Krams, Jean-Paul Aben, Ranil de Silva

**Affiliations:** 1 Vascular Biology, National Heart and Lung Institute, Imperial College London, London, UK; 2 Bioengineering, School of Engineering and Materials Science, Queen Mary University of London, London, UK; 3 Pie Medical Imaging, Maastricht, The Netherlands; 4 Royal Brompton & Harefield Hospitals, Guy’s and St. Thomas’ NHS Foundation Trust, Sydney Street, London, SW3 6NP UK

**Keywords:** Shear stress, Optical coherence tomography, Quantitative coronary angiography, Coronary artery disease, Computational fluid dynamics

## Abstract

An approach to rapid simulation of time-averaged wall shear stress (TAWSS) on 3D geometries created from 3D Quantitative Coronary Angiography (3D-QCA) methodology has been developed, which enables rapid computational fluid dynamic (CFD) shear stress simulation. We compared TAWSS estimated from 3D-QCA-CFD with optical coherence tomography (OCT)-based CFD simulations in coronary arteries. 15 normal and 5 stenotic coronary arteries in instrumented minipigs were studied. 3D arterial geometries were reconstructed from 3D-QCA and OCT using common centrelines and matched axial positions. Identical boundary conditions were used for both methods through directly measured vessel-specific inlet blood velocities. TAWSS was calculated for axially matched segments (n = 80 for normal arteries; n = 160 for stenotic arteries) and in 3 mm/60° sectors. Mean TAWSS simulation times for 3D-QCA and OCT-based CFD were 17.8 min and ~ 1.5 h respectively. There were significant but numerically small differences in TAWSS for normal arteries (-0.21 ± 0.64 Pa [95%CI -1.04,1.46], p < 0.001), and no significant difference for stenotic arteries (-0.39 ± 3.04 Pa [95%CI -6.35, 5.56], p = 0.25). Axial TAWSS profiles along vessel lengths were similar between the two methods. There is a trend of underestimation by 3D-QCA at higher values of TAWSS compared with OCT, due to differences in geometry dimensions. Similar spatial distributions of TAWSS in both normal and stenotic arteries were observed from co-registered TAWSS maps. This study suggests that 3D-QCA-based TAWSS is feasible in both normal and stenotic arteries and that further clinical evaluation of rapid TAWSS from 3D-QCA is warranted, which may facilitate clinical adoption of TAWSS assessment.

**Supplementary Information:**

The online version contains supplementary material available at 10.1007/s10554-026-03649-1.

## Introduction

Locally disturbed blood flow is a major determinant of atherosclerotic plaque initiation and progression [1] and can be quantified by metrics of wall shear stress (WSS) which is the frictional force generated by blood flowing over the endothelial surface. Altered shear stress plays a causal role in the initiation and development of advanced coronary atherosclerotic plaques [2]. Clinical studies show associations between WSS metrics, atherosclerotic plaque progression [3, 4] and adverse cardiovascular events [5–7]. The addition of local WSS assessment to intracoronary imaging derived evaluation of plaque morphology, improves the identification of plaques that are likely to progress and develop high risk features [5, 6, 8, 9]. Landmark analyses from the PREDICTION and PROSPECT studies have demonstrated that CFD-derived WSS metrics are predictive of plaque progression and major adverse cardiovascular events [3, 5, 7]. Costopoulos *et al.* further showed that regions of low WSS and high plaque structural stress are independently associated with plaque progression and compositional changes [10]. Candreva *et al.* demonstrated that angiography-derived WSS and FFR pullback metrics correlate significantly with plaque phenotype in chronic coronary syndrome patients [11]. Recent literature highlights the translational potential of CFD for risk stratification and treatment planning in coronary artery disease [12, 13]. However, despite this evidence base, clinical adoption of WSS assessment into the clinical workflow has been hampered by the time-consuming nature of generating 3D arterial geometries which require segmentation and fusion of intracoronary imaging modalities and coronary angiography as well as the overhead of computational fluid dynamic (CFD) simulations.

Several imaging modalities have been employed to reconstruct coronary artery geometries for CFD-based WSS estimation. QCA is widely accessible in clinical practice. The development of 3D-QCA has enabled rapid, automated reconstruction of coronary geometries from standard angiograms, facilitating real-time WSS computation [9, 14]. The main advantages of 3D-QCA are its speed, non-invasiveness, and compatibility with routine clinical workflows. However, its spatial resolution is limited compared to intravascular imaging, and may be affected by vessel overlap, foreshortening, and suboptimal contrast opacification, potentially leading to inaccuracies in lumen geometry and hence WSS estimation [15, 16].

In contrast, OCT provides high-resolution, cross-sectional images of the coronary lumen, allowing for precise characterization of vessel morphology and plaque microstructure [17, 18]. OCT-based CFD is considered the gold standard for WSS estimation due to its superior spatial resolution and ability to detect subtle changes in lumen geometry, particularly in complex or tortuous vessels [19]. However, OCT requires additional intracoronary instrumentation, is more time-consuming, and is less widely available than angiography. Comparative studies have shown that while 3D-QCA tends to slightly overestimate lumen diameters and underestimate WSS compared to OCT, the overall spatial distribution of WSS is similar between the two methods, supporting the potential feasibility of rapid WSS assessment from angiography in clinical settings [15, 18]. Despite these advances, few studies have performed systematic quantitative comparisons of how rapid 3D-QCA derived shear stress values and distribution compare with OCT-based methodology, particularly in anatomically matched segments and using directly measured physiological boundary conditions.

The accuracy of CFD-based WSS estimation depends critically on the fidelity of the reconstructed geometry, the choice of boundary conditions, and the underlying modelling assumptions. Most studies, including the present work, assume blood is an incompressible Newtonian fluid, with density and viscosity values set according to physiological norms [20, 21]. Inlet boundary conditions are typically prescribed using patient-specific or population-averaged velocity profiles, while outlet conditions may use zero-pressure or resistance-based models. Vessel walls are generally modelled as rigid, although recent advances in fluid–structure interaction (FSI) modelling have enabled the incorporation of wall compliance for greater physiological relevance [22]. In this study, we evaluated directly measured vessel-specific Doppler velocity data for inlet conditions, pressure-free outlets, and curvature-based meshing strategies for both 3D-QCA and OCT-derived geometries.

The present study aims to address current knowledge gaps by providing a comprehensive, head-to-head comparison of 3D-QCA and OCT-based WSS estimation in a preclinical model, with a focus on regional WSS distributions, computational workflow, and demonstrating potential for clinical translation. We undertook an experimental study to evaluate a novel approach with the potential to provide real-time estimation of time averaged wall shear stress (TAWSS) from arterial geometries generated by 3-dimensional quantitative coronary angiographic (3D-QCA) analysis of coronary angiograms. We compared this method of TAWSS quantification with our previously reported optical coherence tomography (OCT)-based method [2] in both normal and stenotic coronary arteries. We provide data which suggest that 3D-QCA based TAWSS calculation is feasible, can be completed in less than 20 min and provides quantitative outputs and regional TAWSS maps that align with OCT-based TAWSS analyses.

## Materials and methods

### Animal studies

Experimental studies were performed in transgenic *D374Y*-PCSK9 hyperlipidaemic minipigs (n = 5) fed a cholate free high-fat-high-cholesterol diet (Test Diet) as previously reported [2]. The use of *D374Y-PCSK9* hyperlipidemic minipigs is appropriate for modeling atherosclerotic disease due to their genetically induced dyslipidemia and consistent development of advanced coronary plaques that closely resemble human pathology. These minipigs exhibit key features of human-like atherogenesis, including lipid-rich plaques, fibrous cap formation, and endothelial dysfunction, making them highly suitable for translational research [23]. Compared to other animal models such as rodents, the *D374Y-PCSK9* minipig model offers superior anatomical and physiological similarity to humans, enabling intravascular imaging and interventional procedures with high fidelity. Furthermore, this model develops spontaneous and progressive coronary atherosclerosis which can be accelerated within a practical timeframe, facilitating the evaluation of therapeutic strategies and imaging modalities in a clinically relevant setting. Before cardiac catheterisation procedures, animals were initially premedicated with oral diazepam (1 mg/kg) and acepromazine (1 mg/kg) after which intramuscular ketamine (5 mg/kg) and xylazine (1 mg/kg) was administered. After endotracheal intubation, animals were mechanically ventilated with isoflurane (1–3%) as an inhalational anaesthetic. Vital signs including end-tidal CO2, oxygen saturations, arterial blood pressure and electrocardiogram were monitored continuously. At each instrumentation, animals received 1 g ampicillin intravenously. The day before stenotic stent implantation, animals received clopidogrel 600 mg and aspirin 300 mg. Following stenotic stent implantation, animals received daily aspirin 75 mg and clopidogrel 150 mg orally for the study duration.

Cardiac catheterisation was performed via an 8 French right femoral sheath (Arrow International) placed percutaneously under ultrasound guidance. Unfractionated heparin (5000u) was administered with a further 1000u administered every hour. The left and right coronary arteries were engaged with an 8 F Hockey Stick I guiding catheter. Coronary angiography was performed using a single plane X-ray fluoroscopy system (Innova 4100, GE Healthcare). Angiograms were acquired after administration of intracoronary isosorbide dinitrate (0.1–0.3 mg) in orthogonal views (> 30 deg separation), using standard angiographic projections for porcine procedures including anteroposterior, left anterior oblique (LAO 30 deg lateral) and lateral views, with views selected to avoid vessel overlap. Respiration was briefly held in end-expiration to minimise respiratory motion artefact. Angiograms were acquired using iodinated contrast (iodixanol 320 mgI/mL, GE Healthcare, UK). Inlet coronary blood velocity was measured in each coronary artery at proximal locations using a Combowire (Volcano Corporation, San Diego, CA, USA). Equalisation of pressure was performed at the guide catheter tip. Measurements were undertaken > 5 mm from a significant sidebranch (> 2 mm diameter) for 10–20 cycles. Intracoronary frequency domain OCT was performed with an Illumien system and Dragonfly-2 catheters (Abbott Vascular). OCT pullbacks were performed at 20 mm/s during pump injection of iodixanol (4–8 mL/s) and location of the OCT catheters were documented by angiography. Following completion of baseline angiography, Combowire and OCT, a modified 3 × 15 mm PK Papyrus stent (Biotronik) was placed in the right coronary artery (RCA, n = 4) and left circumflex coronary artery (LCx, n = 1) to create a coronary stenosis [24]. The severity of stenosis was not predefined, as the modified stent design induces variable degrees of luminal narrowing depending on vessel anatomy. Coronary angiography, Combowire and OCT studies were repeated after the implementation of the stent at baseline. Collectively, 15 normal (n = 5 LAD, n = 5 LCx, n = 5 RCA) and 5 stenotic arteries were imaged. All animal procedures were conducted in accordance with institutional guidelines of the Griffin Institute, London, UK and the Guide for the Care and Use of Laboratory Animals (Institute of Laboratory Animal Resources, 1996). All experimental procedures received the approval of the UK Home Office (PPL 70/7765).

## Reconstruction of 3D coronary arterial geometries and CFD for WSS computation

### 3D-QCA

**Fig. 1:**
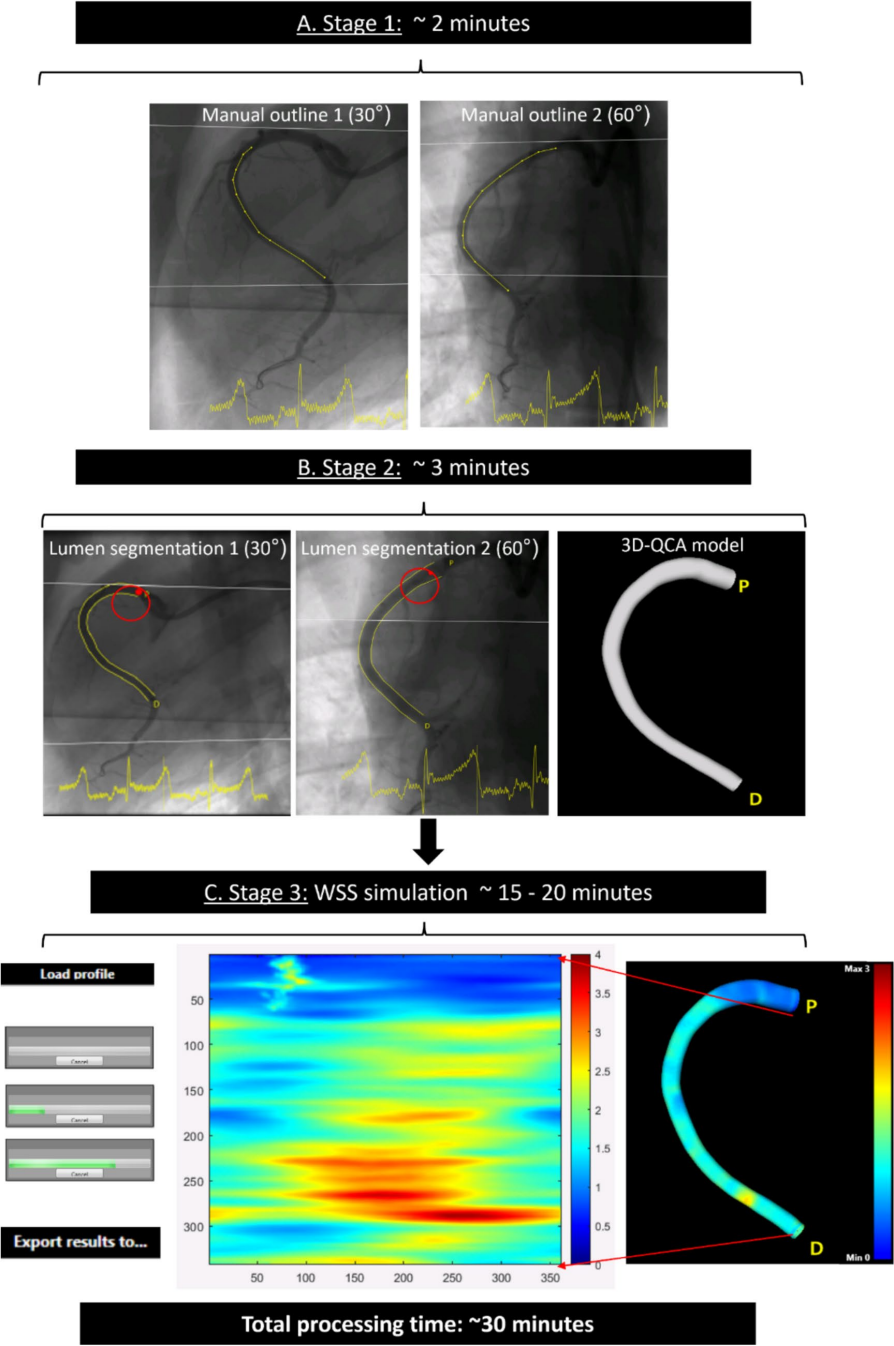
3D-QCA-based vessel reconstruction and framework (CAAS WSS 8.2 software). Workflow shows construction of 3D arterial geometries using orthogonal angiogram frames and generation of TAWSS maps. 3D-QCA: 3-dimensional quantitative coronary angiography; TAWSS: time-averaged wall shear stress

The workflow for computation of the TAWSS from 3D-QCA using prototype software CAAS Workstation WSS 8.2 (Pie Medical Imaging, the Netherlands) is summarised (Fig. 1)*. 3D-QCA* reconstructions were created using two angiographic projections with a well opacified vessel lumen, side-branches visible and a difference in angulation of more than 30 degrees. The coronary segment of interest as reconstructed by 3D-QCA was defined from OCT by matched anatomical landmarks present in OCT pullbacks such as the proximal inlet, major side-branches and stent edges. Points were manually identified along the centre of the vessel in each projection to generate the vessel centreline, and the software performed automated contour detection of the luminal borders. Manual corrections were applied in cases of contour irregularities, poor contrast opacification, or vessel overlap, and a common image point was indicated at the same anatomical location in both projections to enable co-registration. The coronary segment was then reconstructed in three-dimensional space assuming an elliptical cross-section shape, and the 3D mesh geometry was constructed for CFD analysis.

In each angiographic projection end-diastolic frames, adequately filled with contrast, were automatically selected by matching to the electrocardiogram. Points were manually identified along the centre of the vessel, creating a centreline along the coronary segment (Fig. 1A) and the software performed a contour detection of the luminal borders [25]. When needed, contour correction was performed. A common image point was indicated at the same anatomical position in both projections for co-registration (Fig. 1B) and the coronary segment of interest was automatically reconstructed in three-dimensional space assuming an elliptical cross-section shape [26]. The 3D mesh geometry was then constructed (3D model) and CFD was computed within the CAAS WSS software, for which boundary conditions are detailed below under CFD analysis.

### OCT

**Fig. 2:**
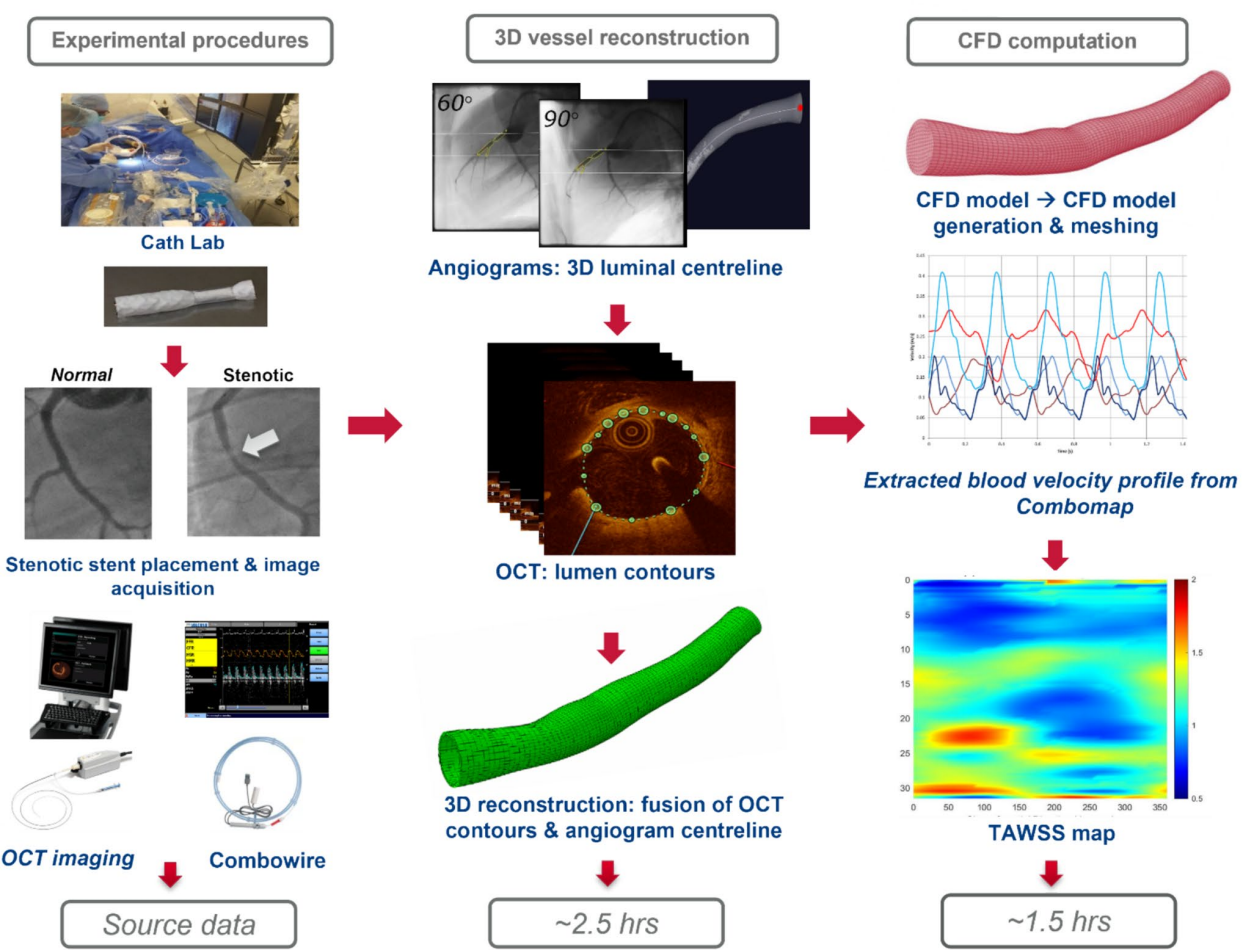
Experimental data acquisition, OCT-based vessel reconstruction and WSS framework. Workflow shows acquisition of source data from minipig experiments (1), which were used to create the 3D arterial geometries (2) for the final computation of TAWSS (3). OCT: optical coherence tomography; TAWSS: time-averaged wall shear stress; WSS: wall shear stress

OCT-based vessel reconstruction methodology was performed as previously reported [2] (Fig. 2). Coronary OCT lumen contours (0.2 mm/frame) were segmented using CAAS IntraVascular Software (Pie Medical Imaging, The Netherlands). Only clear image frames with visible lumen and vessel > 270 degrees continuous arc were analysed. Contour detection was guided by a tracer system, followed by manual delineation of the lumen borders according to the recommendations of the intravascular OCT working group, ensuring anatomical accuracy and consistency across reconstructions. Whenever a side-branch was present in the OCT image, the lumen was segmented as if no side-branch was present. The 3D coronary artery centreline was extracted from each 3D-QCA model using a validated method previously described by Pedrigi et al. [2]. Briefly, the catheter path was reconstructed from orthogonal angiographic frames using CAAS 5.11 software, and the centreline was derived using the Vascular Modelling Toolkit (VMTK). OCT frame contours were then placed along this path using custom Matlab software, which determined frame positions based on pullback speed and aligned contours normal to the centreline using a discrete approximation to the Frenet–Serret formulae. A single rotation angle was calculated for all contours using the out-of-centre vector approach described [27] enabling accurate orientation of each frame. The resulting 3D lumen surface was smoothed in VMTK to generate the final in vivo geometry. This output, together with OCT lumen contours was used to generate the 3D model from 3D OCT-based coronary arterial geometries. A common centreline was used for both 3D-QCA and OCT arterial geometries to enable generation of co-registered shear stress maps.

## CFD analysis

Discretisation of 3D-QCA reconstructions was performed using tetrahedral elements with a minimum element size of 0.05 mm, according to a curvature-based approach and 4 near-wall tetrahedral layers whereas hexahedral elements were used for OCT-derived models. Other than the arterial geometries, all CFD simulation parameters and boundary conditions were identical between the 3D-QCA and OCT methodologies to ensure direct comparability. Flow at the inlet was assigned using directly measured time-varying Doppler velocity data, as described in Sect. "Animal studies", applied using a parabolic profile. The directly measured velocity profiles were inherently vessel specific and so were individual to each of the 15 normal and 5 stenotic geometries studied. Examples of the inlet waveforms can be seen in Fogell et al. 2023 [28]. Inlet and outlet extensions were excluded from all CFD geometries. Vessel outlets were assumed to be pressure-free (0 mmHg). Blood was assumed to be a Newtonian fluid with a density of 1050 kg/m^3 and dynamic viscosity of 0.0035 Pa/s (3.5 mPas). The lumen wall was treated as a rigid, no-slip boundary. In CAAS WSS, the governing equations of fluid motion were solved along the cardiac cycle based on the Kratos Multi-Physics implemented in CAAS Workstation WSS software [29]. Time-varying CFD simulations were run using Abaqus\CFD v6.14 for OCT-based geometries. CFD simulation parameters can be referred to in Supplementary Table 1. All simulations were run on a desktop computer with processor Intel Core i7-3770, 3.40 GHz, 4 cores, RAM 16.0 GB.

## Data analysis and comparison of TAWSS maps

The following approach was used to account for the different number of nodes in the computational meshes used for OCT and 3D-QCA CFD analyses. Anatomically matched vessel segments for paired comparison within the OCT and 3D-QCA geometries were defined, for comparison of TAWSS along the vessel lengths. For axial TAWSS comparison, the vessels were divided along the length of the 3D coronary reconstruction into 80 axial segments per normal artery and 160 axial segments per stenotic artery to increase the resolution of the 3D model for greater accuracy of the stenosis captured in the reconstruction. Within each segment, the TAWSS of both OCT and 3D-QCA was calculated by averaging the TAWSS along the circumference at each centreline position to obtain a representation of how TAWSS varies along each vessel segment of interest. To compare regional distributions of TAWSS between 3D-QCA and OCT-CFD, a method was developed to co-register the TAWSS maps, allowing them to share a common cutline in order to produce co-registered maps. Briefly, the OCT mesh geometry was rotated and translated to be aligned to the 3D-QCA mesh geometry using point-cloud registration. Following alignment, a 3D-QCA cut-line was projected onto the aligned OCT surface mesh geometry and the difference between the nodal position of the original OCT shear map cut-line and projected 3D-QCA cut-line was calculated. This difference was then used to adjust the cutline for each axial position on the OCT model, ensuring the same ‘cut-line’ position between the OCT and 3D-QCA TAWSS maps. For additional quantitative regional comparison, TAWSS values generated by each method were averaged over 3 mm/60° TAWSS sectors.

## Statistical analysis

Numerical variables are presented as median [interquartile range], and categorical variables are presented as absolute values and/or percentages. For nonparametric paired data, analysis was performed using Wilcoxon matched pairs signed ranked tests as well as to compare paired 3 mm/60° averaged TAWSS sectors between OCT and 3D-QCA. For comparisons within the stenotic stent of stenotic arteries, sectors in the proximal and distal region of the stent were included in addition to sectors within the region of maximal stenosis. Bland–Altman analysis was used to calculate the level of agreement between TAWSS estimations through reporting bias and limits of agreement for normal and stenotic vessels. Analyses were performed in GraphPad Prism 9.1.2 (GraphPad Software, California USA) and P values < 0.05 were considered statistically significant.

## Results

3D-QCA and OCT-CFD were performed in normal (n = 15) and stenotic (n = 5) coronary arteries. The 3D-QCA pipeline took a total of approximately 30 min to obtain TAWSS computation for a given vessel (Fig. 1), compared to approximately 4 h for the OCT method per vessel. Mean CFD simulation times were 17.8 min for 3D-QCA, and approximately 1.5 h for OCT-based CFD. There were no significant differences in the lengths of anatomically matched arteries analysed by each method (Supplementary Table 2).

## Comparison of TAWSS magnitudes and axial distributions in normal arteries

**Fig. 3:**
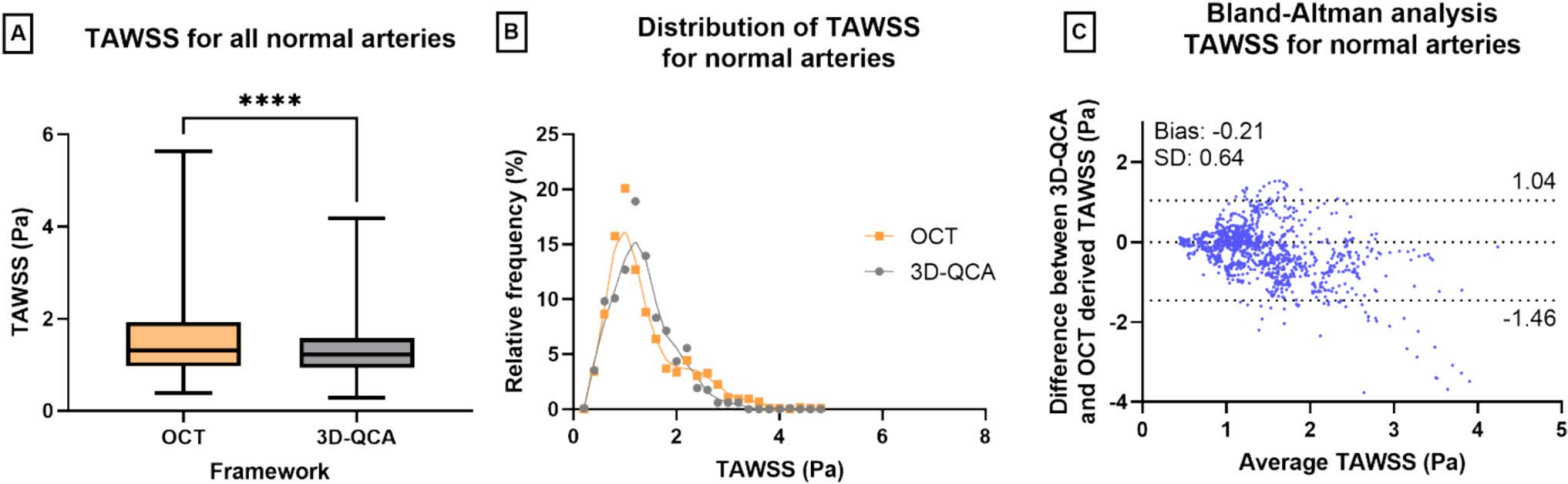
Aggregate analysis of TAWSS data in axially matched segments (n = 1200 paired comparisons) by OCT and 3D-QCA methods from normal coronary arteries (n = 15 arteries). Panel A: Box plot showing median and IQR of TAWSS (Pa) (****P = 0.0002); Panel B: relative frequency distribution of TAWSS (Pa); and Panel C: Bland–Altman analysis showing difference in TAWSS (Pa) vs average TAWSS (Pa)0.3D-QCA: 3-dimensional quantitative coronary angiography; IQR: Interquartile Range; OCT: optical coherence tomography; TAWSS: time-averaged wall shear stress

TAWSS was sampled for 80 axially matched segments along the length of each artery to enable paired comparison of outputs from the 3D-QCA and OCT-models. In an aggregate analysis, although statistically significant differences between the axial segment average TAWSS estimations were observed between the methods for normal vessels (P < 0.0001; 1200 paired comparisons; Fig. 3A), these differences were numerically small (Table 1) with similar distributions of TAWSS determined by 3D QCA and OCT for all normal arteries (Fig. 3B). This was supported by Bland–Altman analysis which shows the agreement between TAWSS estimations for normal arteries, with a mean difference of −0.21 ± 0.64 Pa (95% confidence interval (CI): −1.46–1.04) (Fig. 3C).

**Table 1:** TAWSS estimations by 3D-QCA and OCT-CFD for all normal arteries

Artery	TAWSS (Pa) Median (IQR)		Significance (P value)
	OCT	3D-QCA	
LAD	1.16 (0.85,1.92)	1.25 (0.89, 1.73)	0.0001
LCx	1.44 (1.14, 2.33)	1.31 (0.99, 1.87)	< 0.0001
RCA	1.18 (0.87, 1.69)	1.13 (0.87, 1.33)	< 0.0001
All normal arteries	1.23 (0.94, 1.62)	1.12 (0.88, 1.68)	< 0.0001

*Abbreviations*: 3D-QCA: 3-dimensional quantitative coronary angiography; LAD: left anterior descending artery; LCx: left circumflex artery; OCT: optical coherence tomography; RCA: right coronary artery; TAWSS: time-averaged wall shear stress

## Comparison of TAWSS magnitudes and distributions stratified by individual coronary arteries

**Fig. 4:**
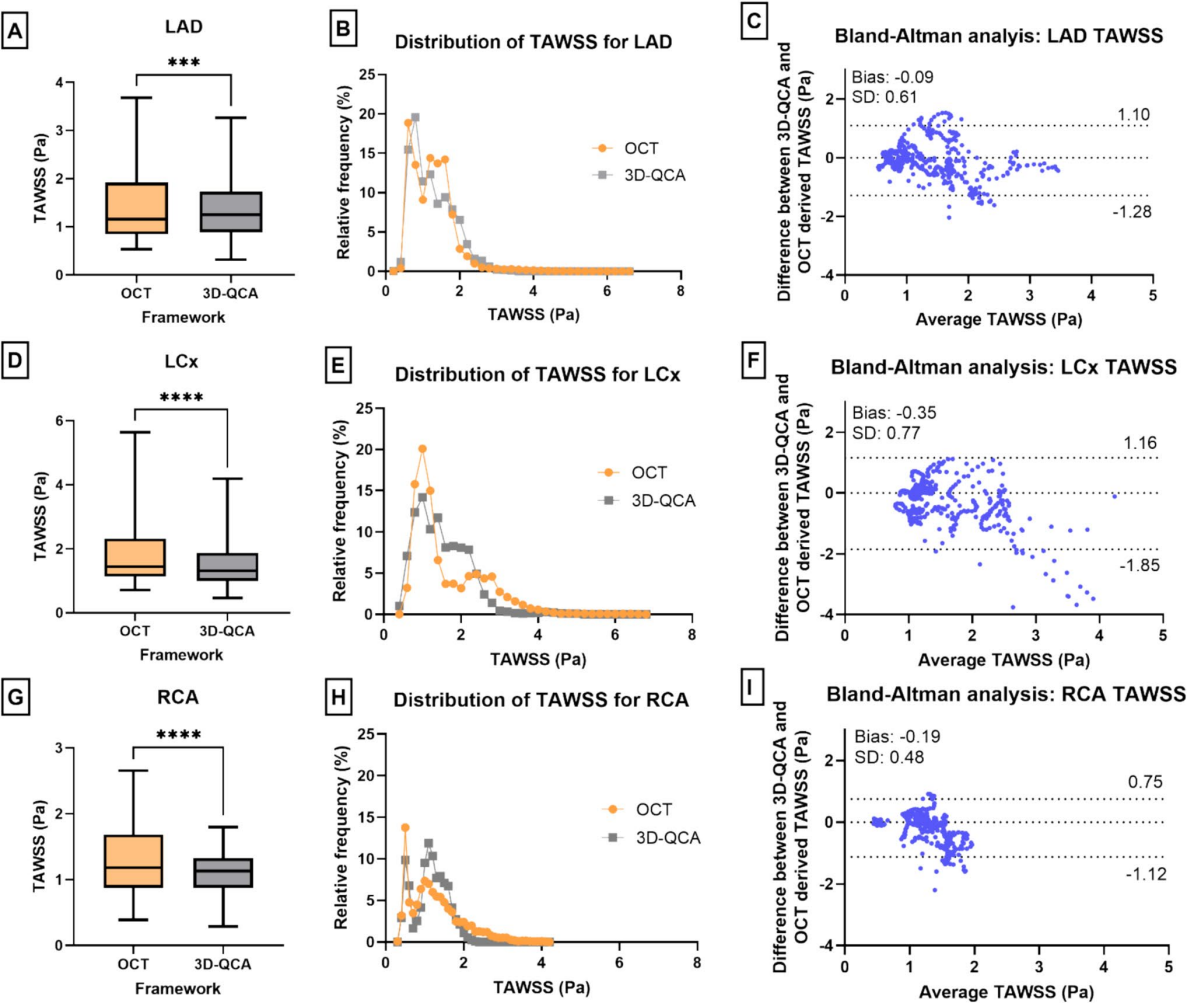
Comparison of TAWSS by OCT-CFD and 3D-QCA—stratified per artery analysis for the LAD (rows A-C, n = 400 paired comparisons), LCx (rows D-F, n = 400 paired comparisons) and RCA (rows G-I, n = 400 paired comparisons). Box and whisker plots showing median [IQR] values of TAWSS (Pa) (panels A, D, E); relative frequency distribution plots of TAWSS (Pa) (panels B, E, H); and Bland–Altman plots (panels C, F, I) of TAWSS (Pa) estimations by OCT and 3D-QCA are shown. *** p = 0.0001; ****p < 0.0001. 3D-QCA: 3-dimensional quantitative coronary angiography; CFD: computational fluid dynamics; IQR: interquartile range; LAD: left anterior descending artery; LCx: left circumflex artery; OCT: optical coherence tomography; RCA: right coronary artery; TAWSS: time-averaged wall shear stress

Comparisons of TAWSS by OCT and 3D-QCA were performed for each coronary artery (Fig. 4, Table 1). The relative frequency distributions of TAWSS computed by each method were similar (Fig. 4). Agreement between the 3D-QCA and OCT methods was best for the LAD (Fig. 4A–C) with the least difference and smallest bias (mean difference: −0.09 ± 0.61 Pa, 95% CI: −1.28–1.10, P = 0.0001). Numerical differences between the OCT and 3D-QCA approaches were greatest for the circumflex artery (mean difference: −0.35 ± 0.77 Pa, 95% CI: −1.85–1.16, P < 0.0001; Fig. 4D-F). Similar differences in 3D-QCA and OCT-derived TAWSS magnitudes and frequency distributions were found for the RCA (Fig. 4G, I). Bland–Altman analysis suggests a small bias in estimating TAWSS for the RCA by 3D-QCA (mean difference: −0.19 ± 0.48 Pa, 95% CI: −1.12–0.75).

## Comparison of TAWSS magnitudes and axial distributions in stenotic arteries

**Fig. 5:**
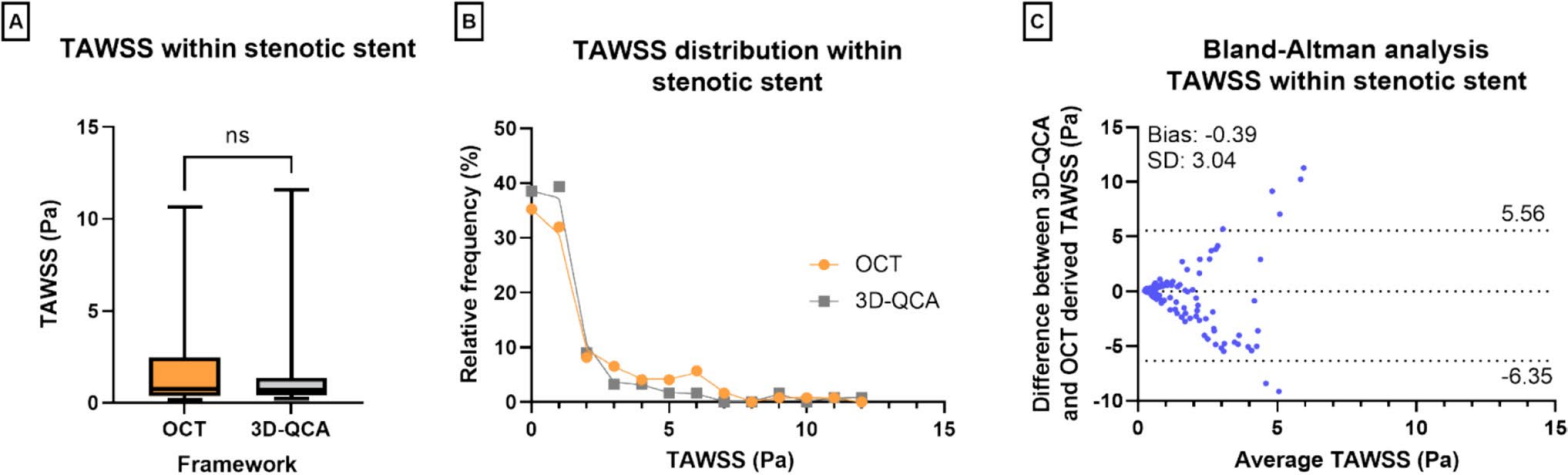
Comparison of TAWSS by OCT-CFD and 3D-QCA in stenotic arteries. Data for the entire artery are shown (plots A–C, *n* = 800 paired comparisons), with a stratified analysis within the stenotic segments (plots D–F, *n* = 200 paired comparisons). Panel A shows box and whisker plots comparing median TAWSS (Pa): OCT: 0.75 [0.36, 2.48] Pa vs. 3D-QCA: 0.68 [0.41, 1.39] Pa (*P* = 0.25). Panel B presents relative frequency distributions of TAWSS (Pa) for both methods. Panel C displays Bland–Altman analysis with a mean difference of –0.39 ± 3.04 Pa and 95% confidence interval of –6.35 to 5.56 Pa, indicating no statistically significant difference. *** *P* = 0.0005. 3D-QCA: 3-dimensional quantitative coronary angiography; CFD: computational fluid dynamics; IQR: interquartile range; OCT: optical coherence tomography; TAWSS: time-averaged wall shear stress.

In a stratified analysis within the stenotic stent segments of stenotic arteries, no statistically significant difference in TAWSS was estimated by OCT and 3D-QCA CFD. A detailed summary of the median values, distributions and agreement analysis is presented in Fig. 5. Differences in absolute TAWSS values can be explained by the differences in measurement of minimal arterial diameter, which are higher by 3D-QCA compared to OCT (Supplementary Fig. 1).

## Comparison of regional TAWSS using co-registered shear stress maps generated from OCT and 3D-QCA models

**Fig. 6:**
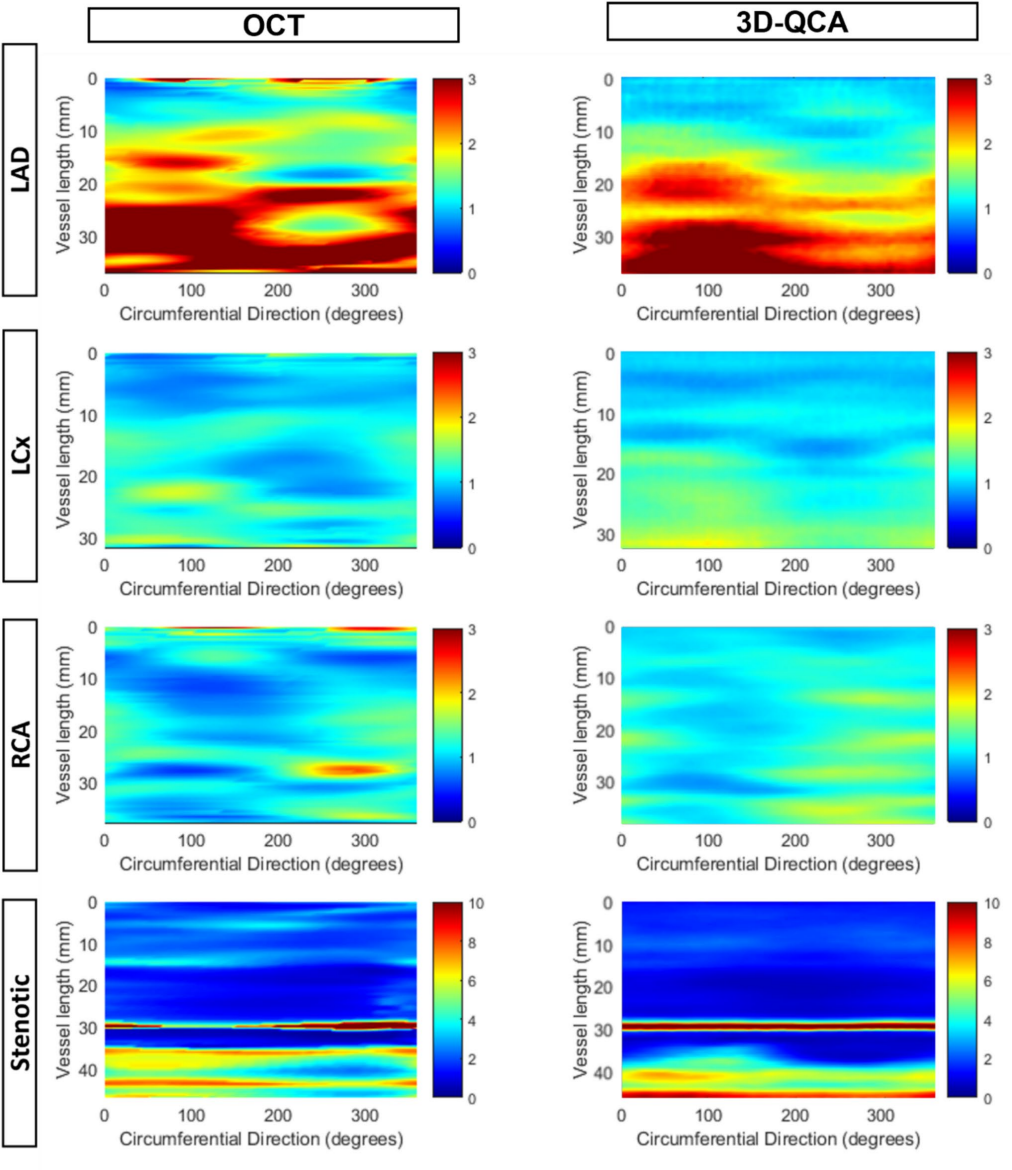
A representative example of co-registered shear stress maps showing regional distributions of TAWSS between OCT-CFD (left column) and 3D-QCA (right column) for LAD, LCx, RCA and stenotic artery. Maps represent the vessel from the proximal (top) to the distal (bottom). y-axis in mm, with the x-axis representing circumferential direction in degrees. TAWSS values are shown in Pascals (Pa). 3D-QCA: 3-dimensional quantitative coronary angiography; CFD: computational fluid dynamics; LAD: left anterior descending artery; LCx: left circumflex artery; OCT: optical coherence tomography; RCA: right coronary artery; TAWSS: time-averaged wall shear stress

**Fig. 7:**
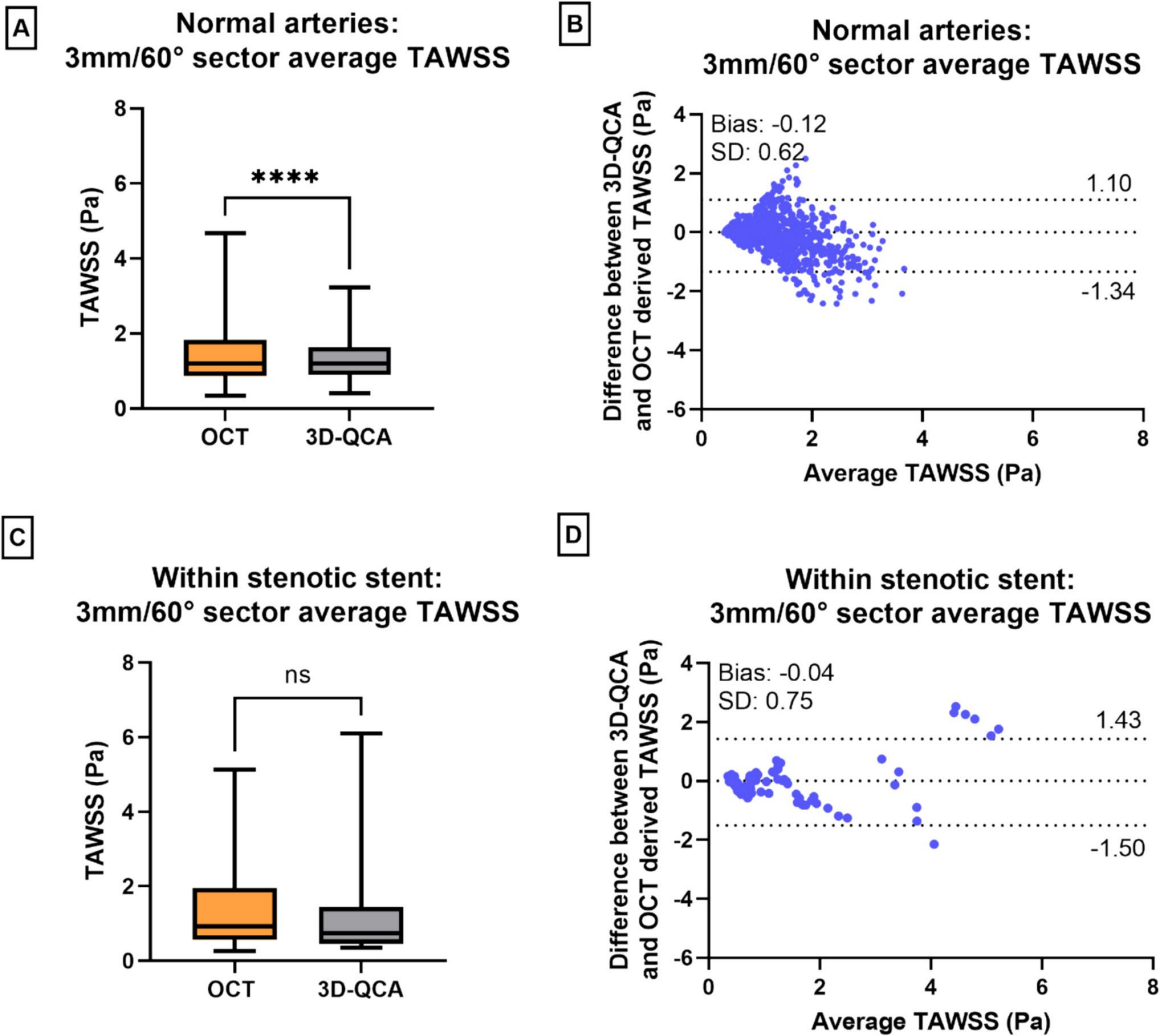
Sector to sector comparison of TAWSS data in circumferentially matched 3 mm/60° sectors in normal arteries (Panels A and C, n = 918 paired comparisons) and stenotic regions (Panels B and D, n = 30 paired comparisons). TAWSS is shown in Pascals (Pa). ****p < 0.0001. TAWSS: time-averaged wall shear stress

Co-registered TAWSS maps demonstrate broadly similar regional distributions of TAWSS in both normal and stenotic arteries (Fig. 6). However, visually there are observable discrepancies in the magnitude and spatial distribution of TAWSS, particularly in stenotic segments. These differences are likely attributable to variations in reconstructed lumen geometry between the two methods, as 3D-QCA tends to overestimate vessel diameter compared to OCT, resulting in lower peak shear stress values. To quantify these differences, we performed quantitative regional circumferential comparisons of TAWSS values generated by OCT and 3D-QCA. For each artery, co-registered maps generated by both approaches were divided into 3 mm/60° sectors (Supplementary Fig. 2). A mean, minimum and maximum TAWSS value was calculated for each sector. In each artery, corresponding sectors underwent paired comparisons (n = 984 sectors for normal arteries and n = 90 for stenotic arteries, which included sectors from the proximal stent, maximal stenosis and distal stent regions). This regional sector analysis showed statistically significant but numerically small differences in TAWSS for normal arteries (OCT: 1.20 [0.86, 1.83] Pa vs 3D-QCA: 1.20 [0.90, 1.64] Pa, P < 0.0001; Fig. 7A, B). There were no statistically significant differences in TAWSS computed within the stenotic stent sectors of stenotic arteries (OCT: 0.92 [0.56, 1.96] Pa vs 3D-QCA: 0.75 [0.46, 1.54] Pa, P = 0.07; Fig. 7C, D). Bland–Altman analysis suggests a small bias in regional sector analyses for normal arteries (mean difference: −0.12 ± 0.62 Pa, 95% CI: −1.34–1.10) and within the stenotic stent segments (mean difference: −0.04 ± 0.75 Pa, 95% CI: −1.50–1.43; Fig. 7B, 7D). 3D-QCA was found to underestimate the maximum TAWSS for both normal and stenotic arteries compared to OCT-based CFD (Supplementary Fig. 3 and 4). The difference in TAWSS between the OCT and 3D-QCA models can be explained by the differences in geometries generated by the two methods as evidenced by the higher mesh diameters in the 3D-QCA models compared to OCT (Supplementary Fig. 1). Similarity in axial TAWSS profiles along the length of vessels supports accurate co-registration of the models generated by each method (Supplementary Fig. 5).

## Discussion

We evaluated a new approach for rapid quantification of TAWSS and its regional distribution using geometries generated by 3D-QCA compared with OCT-CFD methodology in a systematic quantitative experimental study of normal and stenotic coronary arteries. The main findings were as follows: (i) TAWSS values calculated from 3D-QCA and OCT were statistically different but with small numerical differences; (ii) axial profiles of TAWSS along the length of the artery were numerically similar between the two methods and statistically different for normal arteries but not for stenotic arteries; (iii) co-registered regional maps and circumferential sectorial quantification of TAWSS appeared similar; (iv) at higher levels of TAWSS 3D-QCA underestimated TAWSS compared to OCT due to differences in arterial geometry between 3D-QCA and OCT; and (v) mean TAWSS computation times for 3D-QCA compared to OCT-based CFD were significantly lower, being 17.8 min and 1.5 h, respectively. While rapid estimation of TAWSS from 3D-QCA is attractive, whether the numerical differences in TAWSS values are of a magnitude to impact on clinical application of 3D-QCA derived TAWSS estimation requires further investigation.

Generation of accurate 3D coronary arterial geometries underpins CFD simulation for estimation of TAWSS. Previous studies in phantoms, native and stented vessels suggest that measurement of coronary arterial luminal dimensions by OCT is both accurate and reproducible [30–33]. OCT is therefore considered to be reliable for haemodynamic simulations of coronary arteries and provide accurate lumen characterisation along the vessel length [2, 30]. While previous studies indicate that coronary artery lumen dimensions and geometry can be accurately quantified by 3D-QCA [29, 34, 35] we found systematic overestimation of arterial mesh geometry dimensions by 3D-QCA compared with OCT. Inaccurate vessel edge detection due to inhomogeneous opacification of the arteries with iodinated contrast, vessel calibre, suboptimal projection angles, vessel overlap, foreshortening and eccentricity can lead to inaccurate estimation of lumen dimensions [36]. Oversimplification of lumen geometries, vessel tortuosity as well as cardiac and respiratory motion can also affect 3D-QCA derived geometries [35].

The differences in mesh geometries derived from OCT and 3D-QCA translated to systematic differences in TAWSS estimated by each method, with underestimation of TAWSS by 3D-QCA compared with OCT (Fig. 3–4, Supplementary Fig. 2). However, in normal arteries, the difference in absolute TAWSS values between the two methods is numerically small, with Bland–Altman analysis indicating good agreement in the range of TAWSS values of physiological and pathophysiological interest. The Bland–Altman plots show a small mean bias, but relatively wide limits of agreement, particularly in stenotic arteries, suggesting variability in individual sector-level measurements. This variability may reflect differences in local geometry reconstruction and mesh resolution between the two methods. Importantly, the bias toward underestimation by 3D-QCA at higher TAWSS values is consistent with the tendency of angiographic reconstructions to overestimate lumen diameter, which inversely affects shear stress calculations. These findings indicate that while global and median TAWSS values are comparable, caution is warranted when interpreting point-by-point or regional TAWSS values, especially in high-shear regions. Furthermore, the axial distribution of TAWSS along the vessel length appears similar and co-registered TAWSS maps suggest similar regional distributions of TAWSS.

Quantitative comparison of TAWSS values reveal a mean percentage difference of 9.4% in normal arteries and 20.4% in stenotic arteries, with OCT consistently yielding higher values than 3D-QCA. This underestimation by 3D-QCA is reflected in the Bland–Altman analysis, which showed a bias of –0.21 Pa in normal arteries and –0.39 Pa in stenotic arteries. These findings support the presence of a consistent bias toward lower TAWSS values in 3D-QCA-derived simulations, likely due to geometric overestimation of lumen diameter. While the overall distributions and median values were similar, these differences may be more pronounced in regions of high shear stress. Correlation coefficients between OCT and 3D-QCA TAWSS estimations were not reported, which limits direct assessment of linear agreement; however, the comparative analyses and Bland–Altman plots provide insight into the level of concordance between methods.

The present study adds to previous analyses by providing a quantitative investigation of spatial WSS distributions between 3D-QCA and OCT, through assessing regional co-registered TAWSS variations on a local sector-by-sector basis for both normal and stenotic arteries. In addition, artery-specific comparisons for the three main epicardial coronary arteries has been performed systematically, which is important due to variation in curvature and tortuosity. More recently, sectors of low WSS estimated by vessel-type-specific methods have been shown to be associated with significant plaque progression compared with high WSS [37]. 3D-QCA has been previously shown to identify regions of low WSS associated with predictors of atherosclerotic disease progression [9]. Of note, in this study there was a greater level of agreement in distributions and ranges of minimum TAWSS within stenotic stent segments computed by 3D-QCA and OCT-CFD, suggesting that identification of vessel locations with atheroprone low TAWSS may be possible in a rapid timeframe by 3D-QCA-CFD.

Despite the limitations of 3D-QCA, a growing number of studies have sought to derive metrics describing the morphological [38] and haemodynamic [9, 39–42] characteristics of lesions by quantitative analysis of coronary angiograms. Clinical studies have reported assessment of TAWSS using 3D-QCA and shown association with the development of adverse clinical events [6, 43]. Our data further support further clinical evaluation of 3D-QCA derived TAWSS, with a particular benefit of the method presented herein being the rapid image processing and computation times enabling provision of TAWSS data within a timeframe that could fit within the clinical workflow for patients undergoing invasive cardiac catheterisation.

## Clinical implications

TAWSS and its spatial distribution from 3D coronary angiography aligns with those derived from OCT-derived artery geometries for both normal and stenotic coronary arteries. 3D-QCA post processing for quantification of TAWSS can be completed within 30-min per vessel, without need for additional intracoronary imaging techniques or manual image segmentation. This work supports clinical evaluation of TAWSS quantification from 3D-QCA and may facilitate future adoption of TAWSS within the clinical workflow for management of patients with coronary artery disease.

This study was conducted in a controlled experimental setting using a validated preclinical model of coronary atherosclerosis. While this allowed for precise imaging and matched anatomical comparisons between OCT and 3D-QCA, translation to the clinical setting requires consideration of several factors. In clinical practice, image quality may be affected by patient motion, variable contrast opacification, and procedural variability. The angiographic system used in this study had relatively poor spatial resolution compared to modern clinical systems, and we would expect improved image quality and reconstruction in clinical datasets.

The stenotic model used in this study was used to simulate luminal narrowing and generate haemodynamic conditions representative of a clinical coronary artery stenosis. The aim was to induce a reproducible narrowing to study flow disturbances, rather than to model plaque biology. The methodology assessed herein is directly translatable to human clinical evaluations.

## Limitations

This study has a number of limitations. Side branches were not incorporated in the 3D reconstructed models, which could potentially affect assessment of TAWSS distributions. However, it has been shown that incorporation of side branches into 3D geometries only minimally improves the accuracy of final TAWSS predictions [34]. Blood was treated as a Newtonian fluid, as in previous studies [6, 9], which may not accurately approximate pulsatile flow behaviour [43]. These assumptions were made with the aim of reducing computational time and to ensure that identical conditions, other than the geometry and CFD solver, were used for the comparisons. In this analysis, sequential monoplane rather than biplane angiograms were used for analysis. These were analysed while respiration was held to minimise respiratory motion artefacts. In addition, in this study, we only calculated TAWSS rather than other shear stress metrics.

Finally, the sample size of five minipigs limits the statistical power of the study, particularly for subgroup analyses. However, the depth of analysis, including over 900 paired sector comparisons and multiple matched reconstructions, mitigates this limitation. Nonetheless, the findings should be interpreted as preliminary and hypothesis-generating. Larger studies in both preclinical and clinical cohorts are needed to confirm the generalisability and clinical applicability of these results.

## Conclusion

Our data suggest that TAWSS is underestimated by 3D-QCA compared to OCT, though the differences are numerically small. The axial and regional distributions of TAWSS by 3D-QCA appear similar to those derived from OCT in both normal and stenotic arteries. Image processing and computation times were significantly shorter with the 3D-QCA method and completed in a timeframe that could be integrated into a clinical workflow. Whilst there were statistically significant numerical differences in the absolute values of TAWSS generated by each method due to differences in mesh geometry, these differences were numerically small and the similarity in axial and circumferential pattern of TAWSS by each method, together with recent clinical reports [6, 41, 44], suggest the potential utility of this methodology for studying the relationship between altered TAWSS, plaque progression and future clinical events in patients, and advance clinical adoption of TAWSS assessment.

## Supplementary Information

Below is the link to the electronic supplementary material.

Supplementary file1 (DOCX 999 KB)

## Data Availability

No datasets were generated or analysed during the current study.

## Abbreviations

3D-QCA: 3-Dimensional Quantitative Coronary Angiography
CFD: Computational Fluid Dynamics
CI: Confidence Interval
LAD: Left Anterior Descending artery
LCx: Left Circumflex artery
OCT: Optical Coherence Tomography
RCA: Right Coronary Artery
TAWSS: Time Averaged Wall Shear Stress
