# Supplementary material for: Experimental comparisons of optical coherence tomography-based versus angiography-based time-averaged wall shear stress estimations
Source: Int J Cardiovasc Imaging. 2026 Feb 20;42(6):1029–43. doi: 10.1007/s10554-026-03649-1 (PMC13253651; doi:10.1007/s10554-026-03649-1)

**Supplementary Data**

| Parameter | 3D-QCA CFD Simulation | OCT-CFD Simulation | Rationale |
| --- | --- | --- | --- |
| Mesh type | Tetrahedral | Hexahedral | Tetrahedral meshes accommodate angiographic reconstructions with irregular lumen shapes and minimal manual intervention. Hexahedral meshes are feasible for structured OCT geometries. |
| Meshing strategy | Curvature-based adaptive meshing | Structured extruded meshing | Ensures local refinement where curvature is high (e.g., stenoses) and coarser elements in straight segments. |
| Element size (min–max) | 0.05 mm (min), 0.4 mm (max) | 0.05 mm (min), 0.4 mm (max) | Based on prior coronary CFD validation studies to ensure adequate WSS resolution while maintaining computational efficiency. |
| Near-wall layers | 4 | 4 | Improves near-wall resolution and shear stress accuracy. |
| Grid independence | Not performed | Mesh convergence tests demonstrated less than 0.2% difference in mean fluid velocity | A formal grid independence study was not performed. Instead, mesh parameters were fixed across all models, informed by published coronary CFD studies and prior internal experience to ensure consistency and numerical stability. |
| Inlet boundary condition | Time-varying velocity profile from Doppler wire | Time-varying velocity profile from Doppler wire | Vessel-specific waveforms applied to both modalities. Representative profile shown in Supplementary Fig. xxx. |
| Outlet boundary condition | Zero pressure | Zero pressure | Physiological assumption for coronary flow in experimental models. |
| Wall boundary condition | No-slip, rigid wall | No-slip, rigid wall | Standard assumption for comparative analysis of WSS. |
| Solver | Kratos Multiphysics (CAAS WSS) | Abaqus\CFD v6.14 | Choice reflects compatibility with reconstruction pipelines. |
| Time integration | Implicit | Implicit | Ensures stability for pulsatile flow. |
| Time step | 0.005 s | 0.005 s | Selected to satisfy CFL stability and capture velocity waveform dynamics. |
| Simulation duration | 1 cardiac cycle | 1 cardiac cycle | Steady periodic state. |
| Convergence criteria | Momentum & continuity residuals < 1 × 10⁻^6^ |  | Ensures stable and accurate solution. |
| Post-processing | CAAS WSS | MATLAB custom pipeline | Standardized WSS quantification in circumferential sectors. |

**Supplementary Table 1. CFD simulation parameters**

| **Arteries** | **Artery length (mm)** | | **P value** |
| --- | --- | --- | --- |
|  | **OCT** | **3D-QCA** |  |
| **LAD (n = 5)** | 38.1 | 38.0 | **0.19** |
|  | 37.4 | 38.2 |  |
|  | 38.6 | 38.6 |  |
|  | 37.6 | 38.4 |  |
|  | 39.3 | 39.2 |  |
| **LCx (n = 5)** | 33.7 | 33.6 | **0.33** |
|  | 31.4 | 34.8 |  |
|  | 34.2 | 34.6 |  |
|  | 20.0 | 20.0 |  |
|  | 29.2 | 29.0 |  |
| **RCA (n = 5)** | 40.9 | 40.8 | **0.09** |
|  | 36.8 | 37.2 |  |
|  | 39.8 | 40.0 |  |
|  | 39.6 | 39.8 |  |
|  | 40.8 | 41.0 |  |
| **Stenotic (n = 5)** | 37.6 | 37.6 | **0.75** |
|  | 48.0 | 47.8 |  |
|  | 47.4 | 47.6 |  |
|  | 48.7 | 48.8 |  |
|  | 37.7 | 37.6 |  |

_Abbreviations: 3D-QCA: 3-dimensional quantitative coronary angiography; LAD: left anterior descending artery; LCx: left circumflex artery;
OCT: optical coherence tomography; RCA: right coronary artery_ **Supplementary Table 2. Arterial geometry lengths compared by OCT and 3D-QCA**

**Supplementary Figure 1. Bland-Altman analysis of mesh diameters calculated from OCT and 3D-QCA mesh geometries.** Panel A. Aggregate analysis for all normal arteries (n=1200 paired comparisons). Panel B. Aggregate analysis for all stenotic arteries (n=800 paired comparisons). Panel C. Stratified analysis within stenotic segments (n=200 paired comparisons). Panels D-F Stratified analysis by artery for normal arteries (n=400 paired comparisons per artery).

**Supplementary Figure 2. A representative example of co-registered shear stress maps (3mm/60^o^ sectors) showing regional distributions of TAWSS between OCT-CFD (left column) and 3D-QCA (right column) for LAD, LCx, RCA and stenotic artery.** Maps represent the vessel from the proximal (top) to the distal (bottom). y-axis in mm, with the x-axis representing circumferential direction in degrees.

**Supplementary Figure 3.** **Box and whisker plots and Bland-Altman analysis of 3mm/60-degree TAWSS sector-to-sector analysis of minimum (A-B) and maximum TAWSS (C-D) for normal arteries; n=984 paired comparisons each.** **p=0.008, ****p<0.0001.

**Supplementary Figure 4.** **Box and whisker plots and Bland-Altman analysis of 3mm/60-degree TAWSS sector-to-sector analysis of minimum (A-B) and maximum TAWSS (C-D) within the stenotic stent segments; n=90 paired comparisons each.** ***p=0.0001, ****p<0.0001.

**Supplementary Figure 5. Representative examples showing comparison of axial profiles of OCT and 3D-QCA diameter and TAWSS profiles.** Column A shows OCT and 3D-QCA meshes for the LAD, LCX, RCA and stenotic arteries. Column B shows the corresponding average diameter by axial location for each of the arterial geometries. Column C shows the corresponding mean TAWSS for each axial segment.

**Supplementary Figure 6. A representative example of co-registered shear stress maps (3mm/60^o^ sectors) showing regional distributions of minimum TAWSS between OCT-CFD (left column) and 3D-QCA (right column) for LAD, LCx, RCA and stenotic artery**. Maps represent the vessel from the proximal (top) to the distal (bottom). y-axis in mm, with the x-axis representing circumferential direction in degrees.  **Supplementary Figure 7.** **A representative example of co-registered shear stress maps (3mm/60^o^ sectors) showing regional distributions of maximum TAWSS between OCT-CFD (left column) and 3D-QCA (right column) for LAD, LCx, RCA and stenotic artery.** Maps represent the vessel from the proximal (top) to the distal (bottom). y-axis in mm, with the x-axis representing circumferential direction in degrees.


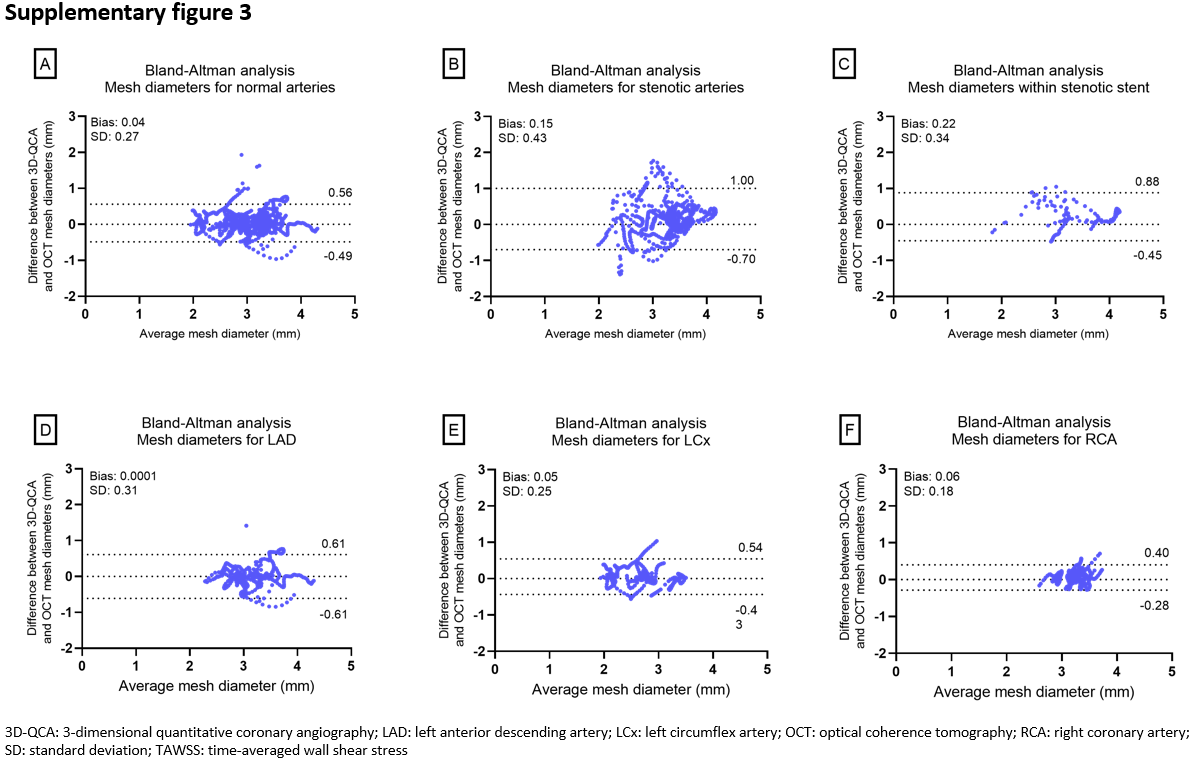


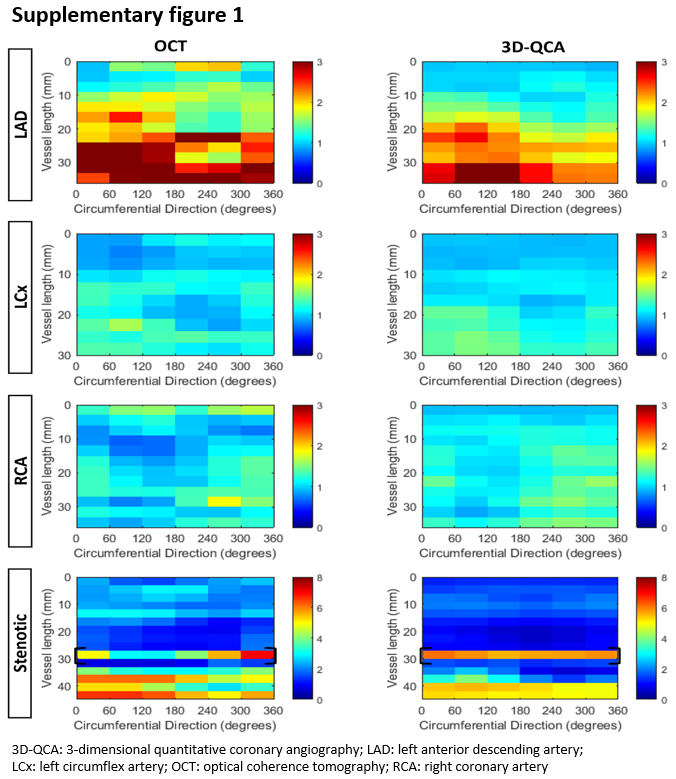


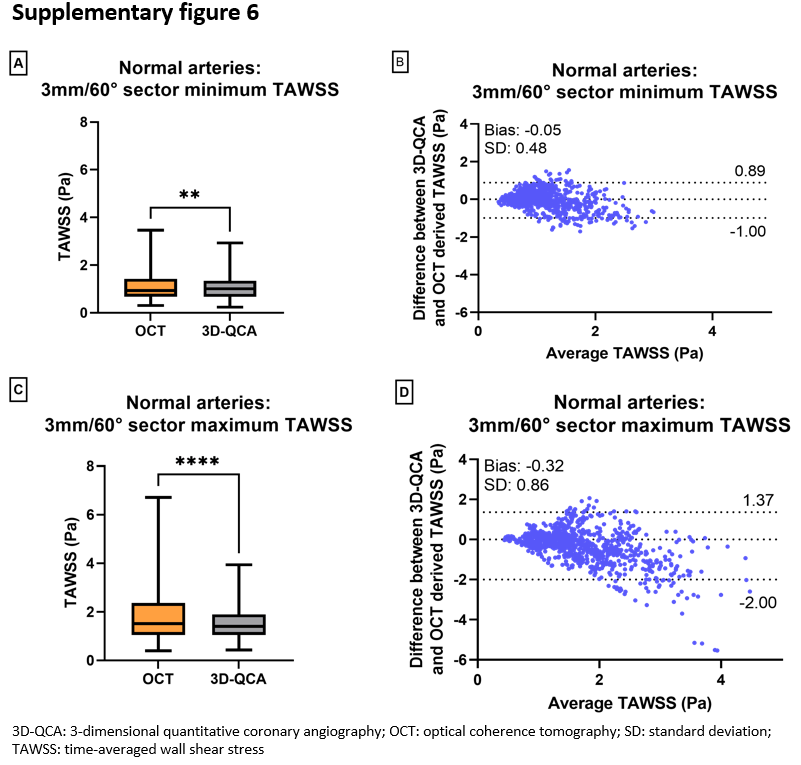


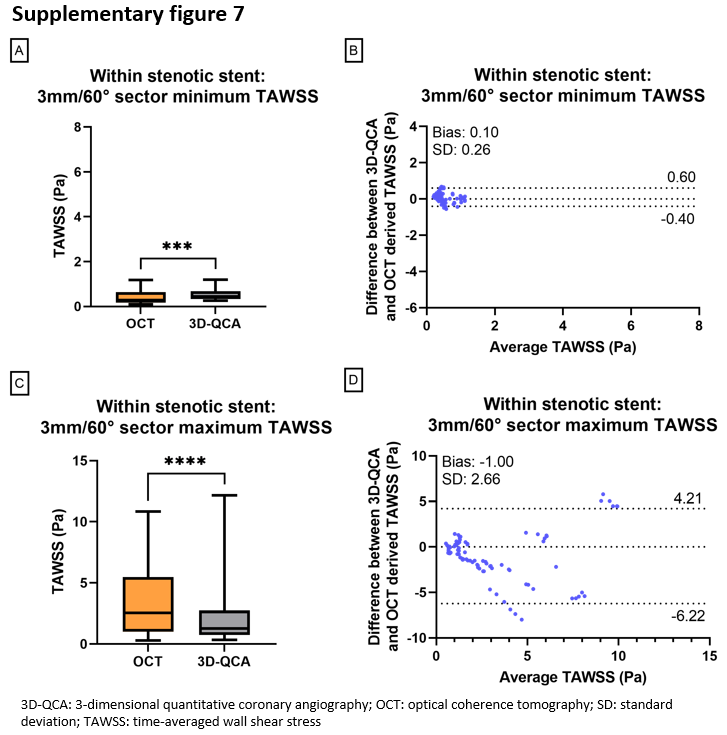


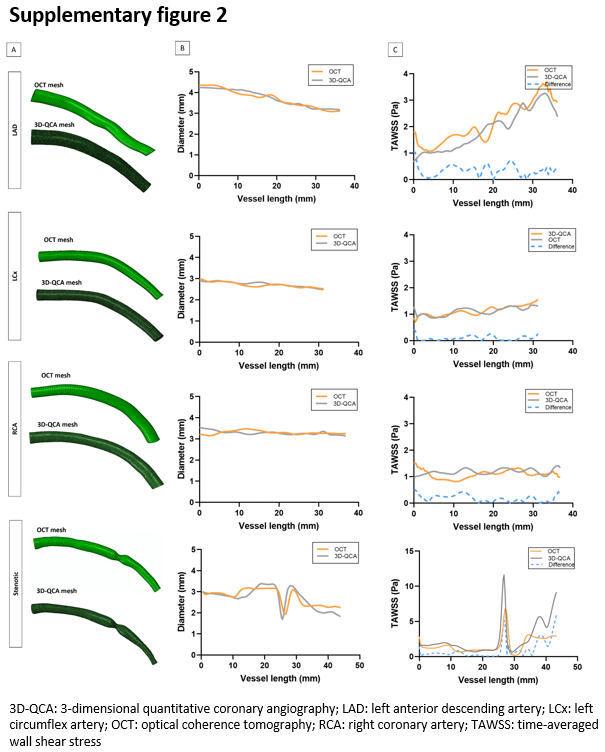


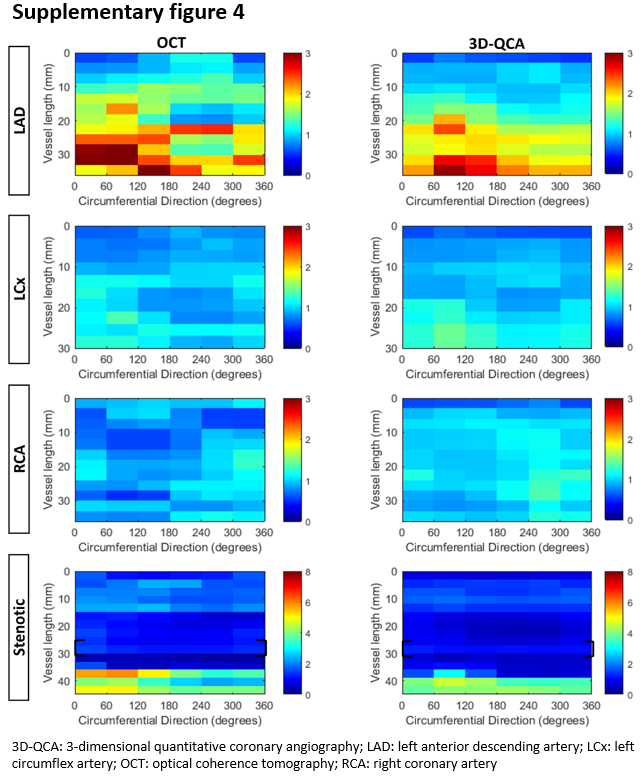


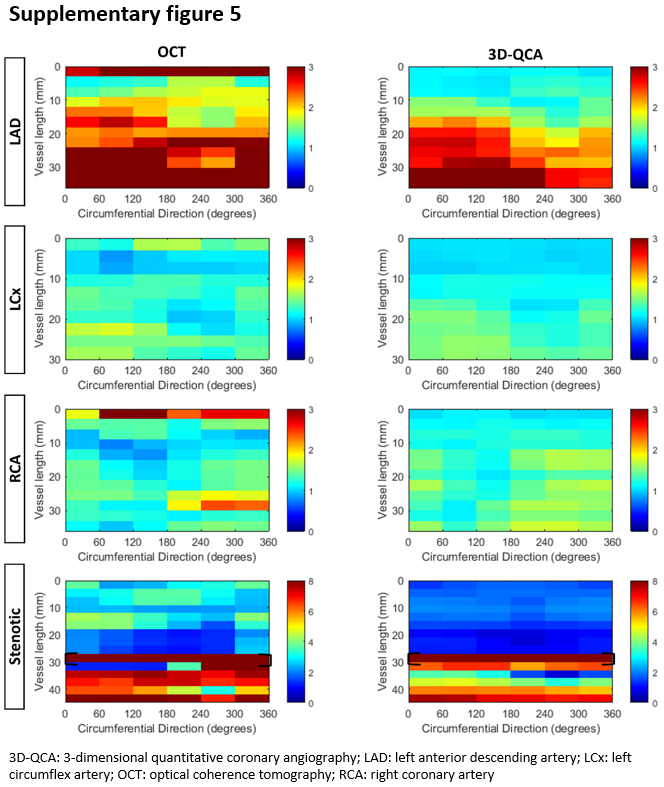

Supplement: Supplementary file 1 — Supplementary file1 (DOCX 999 KB) [file 10554_2026_3649_MOESM1_ESM.docx]
